# Are Patients at Risk for Recurrent Disease Activity After Switching From Remicade^®^ to Remsima^®^? An Observational Study

**DOI:** 10.3389/fmed.2020.00418

**Published:** 2020-08-06

**Authors:** Laixi Xue, K. van Bilsen, M. W. J. Schreurs, M. E. J. van Velthoven, T. O. Missotten, A. A. H. J. Thiadens, R. W. A. M. Kuijpers, P. van Biezen, V. A. S. H. Dalm, J. A. M. van Laar, M. A. W. Hermans, W. A. Dik, P. L. A. van Daele, P. M. van Hagen

**Affiliations:** ^1^Internal Medicine, Erasmus University Rotterdam, Rotterdam, Netherlands; ^2^Academic Center for Rare Immunological Diseases, Erasmus MC, University Medical Center, Rotterdam, Netherlands; ^3^Department of Internal Medicine, Division of Clinical Immunology, Erasmus MC, University Medical Center, Rotterdam, Netherlands; ^4^Department of Internal Medicine, Spaarne Gasthuis Hospital, Haarlem, Netherlands; ^5^Department of Immunology, Erasmus MC, University Medical Centre, Rotterdam, Netherlands; ^6^The Rotterdam Eye Hospital, Rotterdam, Netherlands; ^7^Department of Ophthalmology, Erasmus MC, University Medical Centre, Rotterdam, Netherlands; ^8^Department of Ophthalmology, Albert Schweitzer Hospital, Dordrecht, Netherlands; ^9^Department of Ophthalmology, University Hospital, Vrije Universiteit Brussel, Brussels, Belgium

**Keywords:** infliximab, Remicade^®^, Remsima^®^, CTP 13, biosimilar, rare or orphan immune-mediated inflammatory diseases (IMIDs), anti-TNF-alpha, fucosylation

## Abstract

**Background:** Since the late ‘90s, infliximab (Remicade^®^) is being used successfully to treat patients with several non-infectious immune mediated inflammatory diseases (IMIDs). In recent years, infliximab biosimilars, including Remsima^®^ were introduced in clinical practice.

**Aim:** To investigate the interchangeability of Remicade^®^ (originator infliximab) and its biosimilar Remsima^®^ in patients with rare immune-mediated inflammatory diseases (IMIDs).

**Methods:** This two-phased prospective open label observational study was designed to monitor the transition from Remicade^®^ to Remsima^®^ in patients with rare IMIDs. All included patients were followed during the first 2 years. The primary endpoint was the demonstration of non-difference in quality of life and therapeutic efficacy, as measured by parameters including a safety monitoring program, physicians perception of disease activity (PPDA) and patient self-reported outcomes (PSROs). Secondary outcomes included routine blood analysis, pre-infusion serum drug concentration values and anti-drug antibody formation.

**Results:** Forty eight patients treated with Remicade^®^ were switched to Remsima^®^ in June-July 2016 and subsequently monitored during the first 2 years. The group consisted of patients with sarcoidosis (*n* = 17), Behçet's disease (*n* = 12), non-infectious uveitis (*n* = 11), and other diagnoses (*n* = 8). There were no significant differences in PPDA, PSROs, clinical and laboratory assessments and pre-infusion serum drug concentrations between the groups. De novo anti-drug antibodies were observed in two patients. Seven patients with sarcoidosis and five with another diagnosis developed a significant disease relapse (*n* = 7) or adverse events (*n* = 5) within 2 years; 10 of these patients discontinued Remsima^®^ treatment, one withdrew from the study and one received additional corticosteroid therapy.

**Conclusions:** We observed no significant differences in PSROs, PPDA and laboratory parameters after treatment was switched from Remicade^®^ to Remsima^®^. However, disease relapse or serious events were observed in 12 out of 48 patients when treatment was switched from Remicade^®^ to Remsima^®^. The choice to switch anti-TNF alpha biologics in patients with rare IMIDs, particularly in sarcoidosis, requires well-considered decision-making and accurate monitoring due to a possibly higher incidence of disease worsening.

## Introduction

Remicade^®^ (Janssen Biologics, Leiden, Netherlands) was the first tumor necrosis factor alpha (TNF-alpha)-blocking chimeric monoclonal antibody (the originator infliximab) to be approved by the US Food and Drug Administration (FDA). It was approved in 1998 and later approved by the European Medicines Agency (EMA) in 1999 as a new therapy for Crohn's disease (CD) (1–3). Anti-TNF-alpha therapy rapidly proved to be an efficacious, albeit expensive, treatment for other immune-mediated inflammatory diseases (IMIDs), such as refractory rheumatoid arthritis (RA), ankylosing spondylitis (SpA) and psoriasis (1–3).

Several biosimilars for infliximab have been introduced in the past few years. Remsima^®^ (Celltrion Healthcare, Budapest, Hungary) was the first infliximab biosimilar to be authorized in Europe and was authorized in the USA by the FDA in 2016 for the same therapeutic indications as those approved for Remicade^®^ (4, 5).

Prescribing infliximab biosimilars may represent a good solution for controlling costs. Introducing Remsima^®^ instead of Remicade^®^ as a treatment for IMIDs was estimated to represent a potential savings of 2·89 million to 33·80 million euros in five European countries (Germany, The UK, Italy, the Netherlands and Belgium) over a 1-year period (2015) based on a budget impact model. In 2017, the average drug cost per patient per year was approximately £10.070 for Remicade^®^ compared with £9,063 for Remsima^®^, and these costs are declining further (6, 7).

A few studies have demonstrated that among patients switched from Remicade^®^ to Remsima^®^, Remsima^®^ was either non-different or non-inferior with regard for efficacy and safety among patients with IMIDs who were followed during the first 2 years after switching (6, 8–11). However, non-inferiority studies have been performed only among patients with RA, SpA and IBD, all of which are relatively common IMIDs. Since 2000, infliximab has also been increasingly and successfully used in many rare IMIDs, including non-infectious uveitis, (neuro)sarcoidosis and Behçet's disease (BD) (12–15). To our knowledge, there are no published data on the use of Remsima^®^ as a treatment for these rare diseases. This gap in the knowledge of the field leads to a certain reluctance to prescribe this biosimilar in rare IMIDs even though the prescription of Remsima^®^ instead of Remicade^®^ would significantly diminish treatment costs per patient.

In this study, we evaluated whether the earlier interchangeability of Remicade^®^ and Remsima^®^ reported in patients with more common non-infectious IMIDs would also apply to a cohort of patients with rare IMIDs.

## Materials and Methods

### Study Design and Patients

Given the drug costs of biologics and financial budgets, the department of Clinical Immunology at Erasmus University Medical Center in Rotterdam, The Netherlands, switched 48 patients with rare refractory IMIDs from Remicade^®^ to Remsima^®^ (Table 1). In June 2016, Remicade^®^ was replaced by Remsima^®^ as the standard first choice product in all current and future patients on infliximab. Patients were informed by written information letter and personal communication prior to being switched from Remicade^®^ to Remsima^®^.

**Table 1:** Patients baseline characteristics (*n* = 48).

**AGE (YEAR)**	
Mean ± SD	51 ± 14·7
**Gender**	
Male	46% (*n* = 22/48)
Female	54% (*n* = 26/48)
**DIAGNOSES**	
Sarcoidosis (*n* = 17)	
Behçet's disease (*n* = 12)	
Non-infectious uveitis (*n* = 11)	
Other diagnoses (*n* = 8)	
One systemic sclerosis	
One relapsing polychondritis	
One leucocytoclastic vasculitis	
Two orbital pseudotumor	
One granulomatous common variable Immunodeficiency (CVID)	
One pyoderma gangrenosum	
One spondyloarthritis and vasculitis	
**DURATION OF Remicade^®^ TREATMENT (MONTHS)**	
Mean ± SD	48 ± 38
**FOLLOW-UP TIME (MONTHS) AFTER SWITCHING**	
[Range]	24 [23–25]

Monitoring was designed as a two-phase prospective observational study and approved by the local medical ethical committee (MEC-2016-302).

Patients with rare IMIDs were selected from the outpatient clinic of clinical immunology at the Erasmus University Medical Center (EMC, Rotterdam, The Netherlands). Initially, 50 patients from the outpatient clinic of clinical immunology were eligible for inclusion. The patients had received off-label infliximab treatment because of previously refractory disease and/or unacceptable side effects to standard immunosuppressive agents. Informed consent for off-label Remicade^®^ and Remsima^®^ treatment was obtained from all patients when decided to start infliximab therapy. Two of these patients discontinued infliximab treatment prior to the switch due to clinical remission. Forty-eight patients were included in our monitoring program. The median age at the start of the study population (at the start of the study, June 2016) was 51 years old (SD ± 14·7 years old). The group consisted of patients with sarcoidosis (*n* = 17), BD (*n* = 12), non-infectious uveitis (*n* = 11), and other diagnoses (*n* = 8), as summarized in Table 1.

The inclusion criteria were as follows:

– Stable disease and infliximab treatment for at least 6 months,
– Previous compliance was unremarkable, and
– If applicable, concomitant therapy was not altered for at least 4 weeks prior to inclusion.

After June 1, 2016, all included patients were switched to Remsima^®^ at their first upcoming treatment date. Phase I indicated an intensive monitoring period during the first 6 months of the study (the monitoring phase). The patients were examined and interviewed by the monitoring investigators and by the treating doctors at three time points: at inclusion/baseline (before the first infusion with Remsima^®^) and 3 months after and 6 months after switching. Thereafter, the patients were further routinely examined by their treating doctors as part of their usual care during the follow-up phase (phase II).

The follow-ups performed during the follow-up phase in the first 2 years were conducted at six time points. During phase I, those were on the day of first infusion with Remsima^®^ (baseline score) and three and six months later, and during phase II, those were at 12, 18, and 34 months after the day of inclusion.

Discontinuation of therapy for reasons related to the patient's preference, medical contraindication for continuing biosimilar or total discontinuity of infliximab treatment due to disease activity was permitted at any time point.

### Study Outcomes and Assessments

#### Safety Monitoring Program

Safety monitoring was implemented prior to every treatment infusion at the day care clinic, which is compulsory according to local guidelines. The monitoring consisted of a description of the incidence and type of adverse events, the incidence and type of disease worsening and any decline in physical, psychological and social functions.

#### Physicians Perception of Disease Activity (PPDA)

Objective disease activity was regularly monitored clinically by physicians at the outpatient clinic of the department of Clinical Immunology. During the follow-up period, the physicians were invited to provide descriptive data (remission/stable/active) about disease activity related to the clinical performance of each patient during the past 3 or 6 months. The clinical data obtained in each patient at different time points were provided by the same physician to ensure minimal interobserver variation. The incidence of disease worsening and (adverse) events were calculated based on the full analysis set.

#### Patient Self-Reported Outcomes (PSROs)

PSROs were measured at the three time points (at initiation and after 3 and 6 months) through interviews with patients using the RAND-36 health survey and self-reported disease activity questionnaires. RAND-36 is the Dutch version of the health questionnaire SF-36, which measures the quality of life during the last 4 weeks by nine health dimensions (16).

#### Laboratory Analyses

The secondary outcomes included routine blood assessments, the pre-infusion drug concentration and the presence of anti-drug antibodies (ADA) in serum. Standard clinical blood assessments were conducted prior to every infusion according to the department's protocol for infusions with infliximab. Parameters such as CRP, hemoglobulin, platelets, leucocytes, creatinine, ASAT and ALAT were verified by a physician. Physician approval was needed to start each infliximab infusion.

The infliximab concentration and levels of anti-drug antibodies were analyzed using the pre-infusion serum samples collected at the following three time points: baseline and after 3 and 6 months. Commercially available ELISA-based test systems (MabTrack infliximab) produced by Sanquin (Amsterdam, the Netherlands) were used for measuring infliximab serum concentrations and the levels of anti-drug antibodies. The test systems were used according to the manufacturer's instructions without modification. Cut-off levels of < 0·5 mcg/ml have been proposed in the literature, and a level of 2·8 mcg/ml was considered functional (17). Therefore, pre-infusion infliximab concentrations below 3 mcg/ml and 0·5 mcg/ml were considered low and very low drug concentrations, respectively (17, 18). ADA analyses were performed only in patients with low drug concentrations.

### Statistical Analyses

*The* Wilcoxon one-sample test was applied for comparisons of non-normal distributed descriptive data. A log transformation was applied for all clinical blood parameters, and the mean differences were determined in comparison with the baseline mean. A *p* < 0·05 was considered to indicate a significant difference. A Bonferroni *post hoc* test was applied to calculate the adjusted *p*-value for correction for multiple testing.

## Results

### Safety Monitoring Program

All patients were treated with infliximab for at least 6 months and had shown a stable and lasting clinical response on Remicade^®^. The mean duration of Remicade^®^ treatment prior to the switch was 48 months, and the average follow-up time after switch was 24 months (range, 23 to 25 months) (Table 1). See Supplementary Tables 1–3 for additional background information of patients with sarcoidosis, Behçet's disease and uveitis.

**Figure 1:**
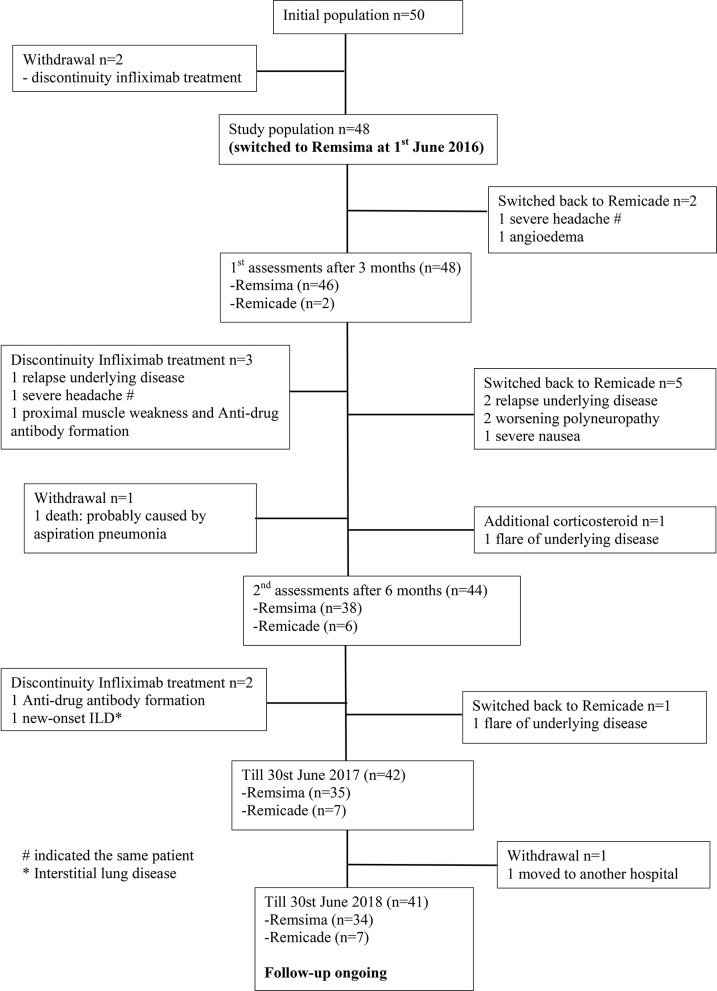
Flowchart study population.

**Figure 2:**
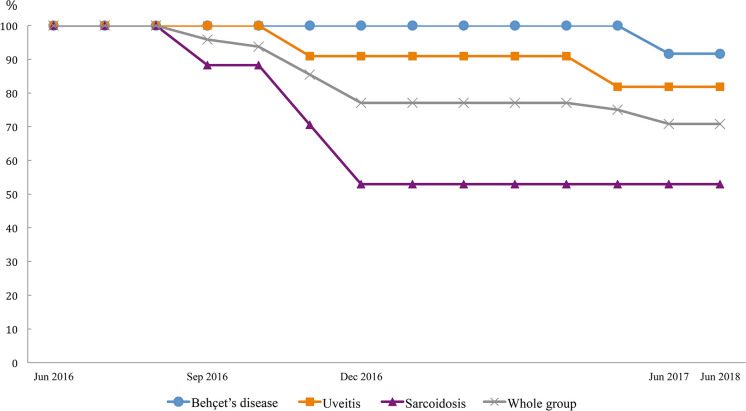
Survival curve of three largest group on Remsima^®^.

All included patients were carefully examined by physicians prior to every treatment infusion and on the clinic day to ensure drug safety. In total, 22 events were observed until June 30st, 2018; these included one death, two significant adverse events, two ADA formation, 7 disease relapses or progression of pre-existing symptoms, two non-related events and eight mild adverse events. Thirteen of these patients received either different treatments (*n* = 12) or additional therapy (*n* = 1) (Figure 1; Table 2). Figure 2 demonstrates the survival curve of Remsima^®^ in the whole group and the three largest groups (sarcoidosis, uveitis and Behçet's disease) in course of time.

**Table 2:** Safety monitoring during the first 24 months.

**Patient**	**Gender**	**Diagnoses**	**Event**	**Treatment modification (Time point)**	**Outcome after treatment modification**
**Death (n** **=** **1)**					
1.	M	Neurosarcoidosis	Probable recurrent aspiration pneumonia	(6th month)	
**Significant adverse events (n** **=** **2)**					
2.	F	Sarcoidosis	Angioedema e·c·i·	Switched back to Remicade^®^ (3rd month)	No more events with angioedema
3.	F	SpA and vasculitis	New onset Interstitial lung disease	Changed over to rituximab (12th month)	Stable on rituximab
***De novo*** **ADA formation (n** **=** **2)**					
4.	F	Sarcoidosis	Proximal muscle weakness in lower limbs +ADA	Discontinued with infliximab (6th month)	Muscle weakness caused by hypocalcaemia and improved after calcium supplementation
5.	M	NeuroBehçet	ADA formation without clinical worsening	Changed over to adalimumab (9th month)	Stable on adalimumab
**Disease relapses (n** **=** **5)**					
6.	M	Neurosarcoidosis	Generalized seizures due to disease relapse	Switched back to Remicade^®^ (6th month)	Stable on Remicade^®^
7.	F	Granulomatous CVID	Generalized seizures due to disease relapse	Changed over to rituximab (6th month)	Stable on rituximab
8.	M	Neurosarcoidosis	Reactivation underlying disease	Switched back to Remicade^®^ (6th month)	Stable on Remicade^®^
9.	F	Relapsing polychondritis	Reactivation underlying disease	Additional corticosteroid (5th month)	Disease in remission after short-term corticosteroid
10.	F	Uveitis	Flare uveitis	Switched back to Remicade^®^ (11th month)	Stable on Remicade^®^
**Progression of pre-existing symptoms (n** **=** **2)**					
11.	M	Sarcoidosis	Worsening of polyneuropathy	Switched back to Remicade^®^ (4th month)	Improvements of symptoms on Remicade^®^
12.	M	Neurosarcoidosis	Worsening of polyneuropathy	Switched back to Remicade^®^ (5th month)	Improvements of symptoms on Remicade^®^
**Other events (n** **=** **2)**					
13.	F	Sarcoidosis	Worsening of headache	1. Switched back to Remicade^®^ (3rd month)Discontinued with infliximab (5th month)	Diagnosed with medication-induced headache, which improved after cessation of painkiller overuse
14	F	Uveitis	Severe nausea	Switched back to Remicade^®^ (5th month)	Persistent nausea caused by diaphragmatic hernia
**Mild adverse events (n** **=** **8): Skin involvements (*****n*** **=** **4), Edema (*****n*** **=** **2), Arthralgia (*****n*** **=** **2)**					

In the first 3 months, two patients on Remsima^®^ switched back to Remicade^®^, one because of progression of pre-existing headache and one because of new-onset angioedema. After this patient switched back to Remicade^®^, angioedema did not reoccur. In the patient suffering from headache, the complaints persisted while on Remicade^®^ treatment and therefore discontinued further infliximab therapy. Eventually, this patient was evaluated by a neurologist and diagnosed with medication-induced headache, which improved after cessation of painkiller use.

Between 3 and 6 months, another five patients had changed back from Remsima^®^ to Remicade^®^ because of relapse of the underlying sarcoidosis (*n* = 2), worsening of pre-existing polyneuropathy (*n* = 2) or severe nausea (*n* = 1). After re-initiation of Remicade^®^, remission was achieved in the patients with disease relapse or worsening of polyneuropathy. Nausea did not improve after the initiation of Remicade^®^. These complaints were probably not disease- or treatment-related but were instead likely caused by diaphragmatic hernia.

During the same period, in one patient with granulomatous disease as a result of common variable immune deficiency (CVID), rituximab was introduced because of disease progression while on Remsima^®^ treatment. One patient with sarcoidosis was diagnosed with proximal bilateral muscle weakness in the lower extremities caused by hypocalcaemia; this was not related to disease activity or Remsima^®^ treatment, and her proximal lower extremity weakness improved after calcium supplementation. However, she discontinued the infliximab due to unexplained clinical symptoms at that moment and *de novo* ADA formation with low serum drug levels.

Additional short-term glucocorticosteroid treatment was administered to one patient with frequent flares of relapsing polychondritis during Remicade^®^ treatment which was comparable after switching to Remsima^®^.

Twelve months after the start of the study, one additional patient showed a flare of uveitis while on Remsima^®^ and was switched back to Remicade^®^ with beneficial effects. Moreover, one patient with a history of recurrent life-threatening neurosarcoidosis had discontinued infliximab treatment and was switched to adalimumab due to *de novo* ADA formation to prevent recurrent disease. One patient with SpA and vacuities was diagnosed with new-onset interstitial lung disease while on Remsima^®^ during the follow-up and was thereafter effectively treated with rituximab therapy.

Finally, there were two cases of withdrawal, including one case of death. This patient suffered from severe therapy-refractory neurosarcoidosis and showed side effects to previously administered disease modifying anti-rheumatic drugs, and he had a history of recurrent aspiration pneumonia; this latter association was probably related to the cause of death. The other patient had chosen to receive further treatment in a hospital near her residency.

Additionally, eight cases with suspected infusion-related mild adverse events, including skin involvement (*n* = 4), ankle oedema (*n* = 2), or arthralgia (*n* = 2), were reported by patients during the follow-up [data not shown].

All signs of relapse of disease activity and significant adverse events were objectively verified and carefully examined by specialized physicians. The cases were broadly discussed with the involved clinical immunologists, neurologists and clinical pharmacologists of Erasmus University Medical Center to find an explanation. Furthermore, the findings were discussed with the supplier, who informed the authorities (LAREB). At that time, no evident association or any explanation between Remsima^®^ and the reported cases was available. Furthermore, the patient suffering from headaches, as well as the patient reporting nausea had persisting symptoms after they were switched back on Remicade^®^, making Remsima^®^ “failure” rather unlikely.

Follow-up is ongoing with 41 patients and 34 of these patients remain on Remsima^®^ treatment.

### Physicians Perception of Disease Activity (PPDA)

**Figure 3:**
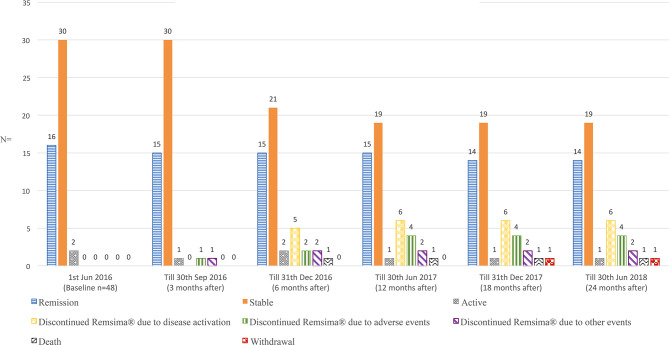
Physicians perception of disease activity on Remsima^®^.

The incidence of increased disease activity scores (*n* = 7) and significant adverse events (*n* = 5) were 14·6% (7/48) and 10·4% (5/48), respectively, based on the full analysis set (*n* = 48) (Figure 3). Supplementary Tables 4, 5, respectively demonstrate the disease activity index measured with Behcet's disease current activity form (19) and Uveitis disease activity index (20) during the first 6 months.

### Patient Self-Reported Outcomes (PSROs)

None of the nine measured quality of life aspects (RAND-36) showed significant differences from baseline among the biosimilar-treated patients during the first 6 months (all *p* > 0·05) (Table 3). The largest difference measured between baseline and 6 months was found in the mental health evaluation. The baseline score ± standard deviation was 74·8 ± 20·0, and the 6th month score ± standard deviation was 77·5 ± 22·1. The calculated Z-score change was −1·970 (*p* = 0·05). Physical functioning demonstrated the smallest Z-score change (−0·18) during the first 6 months, from 65·2 ± 27·4 (baseline ±SD) to 67·3 ± 25·7 (6th month ± SD), *p* = 0·86. However, a further disease subgroup analysis was unreliable due to the limited number of patients in subgroups.

**Table 3:** Results patient self-reported outcomes and clinical blood assessments.

	**Baseline (*n* = 48)**	**3rd month (*n* = 46)**	**6th month (*n* = 38)**	**6th month vs. Baseline**
**RAND-36 dimensions**	**Mean (±SD)**	**Mean (±SD)**	**Mean (±SD)**	**Z-score change**	***P*****-value**
Physical functioning	65·2 (±27·4)	60·5 (±32·8)	67·3 (±25·7)	−0·18	0·86
Social functioning	64·4 (±29·7)	69·1 (±29·0)	68·1 (±29·1)	−0·80	0·42
Role limiting (physical)	40·3 (±36·5)	42·2 (±32·9)	46·1 (±32·3)	−1·20	0·23
Role limiting (emotional)	71·5 (±33·6)	74·5 (±32·5)	76·3 (±28·9)	−0·93	0·35
Mental health	74·8 (±20·0)	75·5 (±23·4)	77·5 (±22·1)	−1·97	0·05*
Vitality	53·5 (±25·8)	51·1 (±22·8)	52·1 (±25·9)	−0·36	0·72
Pain	65·2 (±29·2)	66·6 (±28·5)	63·7 (±30·1)	−0·33	0·74
General health perception	40·2 (±23·5)	36·4 (±22·7)	36·0 (±26·6)	−1·16	0·25
Health change	3·1 (±1·1)	3·3 (±1·1)	3·0 (±1·1)	−0·72	0·47
**Laboratorial parameters**	**Mean (±SD)**	**Mean (±SD)**	**Mean (±SD)**	**Mean difference [95%CI]**	***P-value***
LogCRP	0·51 (±0·61)	0·47 (±0·61)	0·47 (±0·65)	0·040 [−0·094; 0·174]	0·55
LogHb	0·91 (±0·04)^$^^*^	0·91 (±0·05)^$^^*^	0·91 (±0·05)	−0·005 [−0·014; 0·005]	0·31
LogPlatelets	2·40 (±0·12)	2·42 (±0·12)	2·41 (±0·12)	−0·012 [−0·036; 0·012]	0·32
LogLeucocytes	0·81 (±0·12)	0·84 (±0·13)	0·83 (±0·12)	−0·018 [−0·041; 0·005]	0·13
LogCreatinine	1·92 (±0·12)	1·91 (±0·13)	1·90 (±0·14)	0·018 [−0·011; 0·047]	0·22
LogASAT	1·41 (±0·15)	1·42 (±0·13)	1·42 (±0·15)	−0·005 [−0·059; 0·048]	0·84
LogALAT	1·39 (±0·22)	1·39 (±0·21)	1·40 (±0·22)	−0·016 [−0·074; 0·041]	0·57
**Drug concentration and ADA's**	**n/N (%)**	**n/N (%)**	**n/N (%)**		
Concentration <3 mcg/ml^#^	20/48 (41·7)	23/48 (47·9)	24/48 (50·0)		
Concentration <0·5 mcg/ml	13/20 (65·0)	10/23 (43·5)	10/24 (41·7)		
Presence of ADA's	6/13 (46·2)	7/10 (70·0)	8/10 (80·0)		

$ *LogHb (3rd month vs. baseline): Mean difference [95% CI] was −0·008 [−0·014; −0·001] with P = 0·019*.

* *and bold values: Both p-values are no more significant after correction for multiple testing using Bonferroni post hoc test*.

# *Based on full analysis set*.

### Laboratory Analyses

The 95% CI of the mean differences between the groups for all clinical parameters crossed zero with an overall *p* > 0·05 (Table 3). The hemoglobulin level obtained during the first 3 months of Remsima^®^ therapy was significantly higher than the baseline level. The mean difference (baseline vs. 3rd month) was −0·008 mmol/l with 95% CI [0·01; 0·014], *p* = 0·019. The difference was, however, no longer significant after correction for multiple testing (adjusted *p* < 0·001 needed for significance).

Low pre-infusion drug concentrations were found in 20 patients at the time of inclusion, but this finding was not always clinically related to disease activity (Table 3). Six of these patients showed pre-existing ADAs in clinically quiescent disease. All of these patients had a very low drug concentration below 0·5 mcg/ml. Four additional patients developed a low pre-infusion drug concentration during the first 2 years after switching and showed no disease relapse. *De novo* ADA formation was observed in two additional patients without clinical worsening caused by ADA formation at that moment. However, both patients had a fragile clinical condition. Therefore, it was initially decided to discontinue further infliximab treatment in both patients to prevent severe outcomes.

## Discussion

This prospective observational monitoring study demonstrated that the differences in PSRO's and other laboratory parameters between Remsima^®^ and Remicade^®^ treatment groups containing patients with rare IMIDs were non-significant. However, 41% of sarcoidosis patients had significantly increased disease activity or adverse events mainly during the first year after the switch that led to a therapeutic intervention.

In the present study, 34 out of 48 patients with rare IMIDs (70%) who switched from Remicade^®^ to Remsima^®^ maintained this treatment for at least 2 years. Fourteen major events were observed during this period, and these led to alterations in therapy. Previous studies have demonstrated that a safe transition to Remsima^®^ from Remicade^®^ is possible and that Remsima^®^ is non-inferior to Remicade^®^ in common IMIDs, including RA, IBD and SpA. In those studies, approximately 60% of the patients with common IMIDs maintained Remsima^®^ treatment without major or severe adverse events (8, 10, 21, 22).

In contrast, when we evaluated our results regarding the transition from Remicade^®^ to Remsima^®^ in rare IMIDs with those obtained in other reports on patients with more prevalent IMIDs, we found that a substantial number of patients who were diagnosed with granuloma, including (neuro)sarcoidosis, had therapy failure or exacerbation of neurological symptoms that could not be explained by pre-infusion drug levels. These patients could safely return to Remicade^®^ without altering the infusion dosage or interval. In-depth discussions with various specialists initially provided no direct clue about the relevant disease activity.

However, over time, more data have become available supporting a hypothesis to explain the differences observed between Remicade^®^ and Remsima^®^ in the predominantly granulomatous disorders evaluated in this study.

The NOR-SWITCH study demonstrated that there was an almost significant difference for recurrent disease in CD, which is also proposed to be a granulomatous disorder, after patients were switched from Remicade^®^ to Remsima^®^. These results are especially interesting when compared with those obtained for ulcerative colitis (UC) in the same study (8).

Several studies have described the differences in the glycosylation profile between Remicade^®^ and its biosimilars, such as Remsima^®^/Inflectra^®^ and Renflexis^®^/Flixabi^®^ these differences could potentially lead to differential biological actions (23, 24). With regard for infliximab action, the terminal glycan composition of the Fc region plays a critical role in the binding affinity of infliximab to the Fcγ receptor (FcγR) on immune cells, such as natural killer (NK) cells and CD8+ lymphocytes. This binding interaction is essential to induce antibody-dependent cell-mediated cytotoxicity (ADCC) and apoptosis in target cells. *In vitro* data have shown that there is a significant difference in the afucosylated glycoform level in Remicade^®^ (10·0%) compared to Remsima^®^ (6·2%) (*p* < 0·001). This variation in afucosylation levels subsequently leads to lower levels of the FcyR-binding affinity of Remsima^®^ (77·0%) compared to Remicade^®^ (100·9%) and substantially lower ADCC activity for Remsima^®^ (50·3%) compared to Remicade^®^ (99·8%) (*p* < 0·001) (23, 24).

Hence, we postulate that the differences in afucosylation between Remicade^®^ and Remsima^®^ might be associated with the different biological activities of Remsima^®^ in patients with granulomatous diseases, such as sarcoidosis.

This hypothesis is further supported by the finding that sarcoidosis relapses in patients on etanercept (ETN) therapy (25–27). ETN does not bind to membrane-expressed TNF and is therefore unable to induce cytotoxicity by CD8+ and NK cells (28–30). This effect is thought to be necessary to induce a clinical effect in diseases characterized by granuloma formation (28–30). If an afucosylated Fc fragment is also necessary to induce a therapeutic response in non-infectious uveitis is unknown, this finding would be of interest because ETN therapy is associated with relapse of uveitis (31).

We are not aware of other studies that describe differences in clinical efficacies and side effects after switching from Remicade^®^ to Remsima^®^ among patients with rare IMIDs, in particular sarcoidosis. However, it is important to note that we did not had a control arm treated with the originator. Therefore, we could not rule out the possibility that the recurrent disease activity might be a part of the course of disease rather due to the switch to Remicade^®^. We could only include a limited number of patients in our study resulting in a weak statistical power in the subgroups. Moreover, in this study, our objective evaluation of disease activity was based solely on physician perception and may therefore have been affected by differences in interpersonal interpretations.

This is the result of the heterogeneity of the diseases entities included in the study, as well as the lack of standardized activity scores for many of those rare IMIDs. Registries for monitoring patients with rare IMIDs may contribute to draw stronger conclusions.

In conclusion, the implementation of Remsima^®^ treatment among rare immune diseases in particular (neuro)sarcoidosis patients previously treated with Remicade^®^ requires a well-considered decision-making and intensive monitoring due to a possibly higher incidence of disease worsening or exacerbation of (neurologic) symptoms. Remicade^®^ and its biosimilar Remsima^®^ might exert different biological actions in certain disease conditions such as sarcoidosis, and these might be related to differences in fucose content of the Fc fragment. Further studies should be implemented to investigate the biological activity of Remsima^®^, especially in granulomatous diseases such as sarcoidosis. In general, switching from one biologic to another requires careful monitoring to prevent the patient from unwanted side effects or relapse of the disease.

## Data Availability Statement

The datasets generated for this study are available on request to the corresponding author.

## Ethics Statement

The study was approved by the Bioethics Committee of the Erasmus University Medical Centre (MEC-2016-302). The patients/participants provided their informed consent to participate in this study.

## Author Contributions

LX, KB, VD, JL, PD, and PH conceived and designed the study. LX, KB, and PD analyzed and interpreted the data. LX and KB draft the manuscript. All authors contributed to data collection and approved the final manuscript.

## Conflict of Interest

PH was consultant of Abbvie Inc. The remaining authors declare that the research was conducted in the absence of any commercial or financial relationships that could be construed as a potential conflict of interest.
