# Supplementary material for: Are Patients at Risk for Recurrent Disease Activity After Switching From Remicade® to Remsima®? An Observational Study
Source: Front Med (Lausanne). 2020 Aug 6;7:418. doi: 10.3389/fmed.2020.00418 (PMC7424016; doi:10.3389/fmed.2020.00418)
Supplement: Supplementary file 1 [file Data_Sheet_1.docx]

**Supplementary table 1. Baseline characteristics of patients with Behcet’s diesease (n=12)**

| **Age (year)**  Mean ± SD | 52 ± 13·6 |
| --- | --- |
| **Gender**  Male  Female | 58% (n=7/12)  42% (n=5/12) |
| **CNS involvement** | 25% (n=3/12) |
| **Ocular involvement**  Posterior uveitis  Panuveitis  Unspecified | 50% (n=6/12)  n=1  n=2  n=3 |
| **Disease duration (year)**  Mean ± SD | 18 ± 10·1 |
| **Previous treatments**  Adalimumab  Rituximab  Etanercept  Interferon alpha  Intravenous immunoglobulin  Colchicine  Steroids  Cyclosporine  Methotrexate  Thalidomide  Mycophenolate mofetil  Hydroxychloroquine  Pentoxifylline  Azathioprine  Sulfasalazine | 25% (n=3/12)  8% (n=1/12)  8% (n=1/12)  17% (n=2/12)  8% (n=1/12)  75% (n=9/12)  67% (n=8/12)  50% (n=6/12)  42% (n=5/12)  33% (n=4/12)  25% (n=3/12)  25% (n=3/12)  17% (n=2/12)  17% (n=2/12)  8% (n=1/12) |
| **Duration of Remicade^®^ treatment (year)**  Mean ± SD | 4·8 ± 4·1 |

| **Supplementary table 2. Baseline characteristics of patients with Sarcoidosis (n=17)**   \| **Age (year)**  Mean ± SD \| 49 ± 13·3 \| \| --- \| --- \| \| **Gender**  Male  Female \| 59% (n=10/17)  41% (n=7/17) \| \| **CNS involvement** \| 41% (n=7/17) \| \| **Ocular involvement**  Anterior uveitis  Panuveitis  Unspecified \| 24% (n=4/17)  n=2  n=1  n=1 \| \| **Disease duration (year)**  Mean ± SD \| 7·6 ± 2·9 \| \| **Previous treatments**  Adalimumab  Rituximab  Anti-interleukin-6 therapy  Steroids  Hydroxychloroquine  Methotrexate  Azathioprine  Mycophenolate mofetil  Thalidomide \| 24% (n=4/17)  6% (n=1/17)  6% (n=1/17)  88% (n=15/17)  53% (n=9/17)  41% (n=7/17)  29% (n=5/17)  18% (n=3/17)  6% (n=1/17) \| \| **Duration of Remicade^®^ treatment (year)**  Mean ± SD \| 3·2 ± 1·8 \| |
| --- | --- | --- | --- | --- | --- | --- | --- | --- | --- | --- | --- | --- | --- | --- |

**Supplementary table 3. Baseline characteristics of patients with non-infectious uveitis (n=11)**

| **Age (year)**  Mean ± SD | 49 ± 15·7 |
| --- | --- |
| **Gender**  Male  Female | 27% (n=3/11)  73% (n=8/11) |
| **Classification**  Intermediate uveitis  Posterior uveitis  Panuveitis  Unspecified | n=1  n=4  n=3  n=3 |
| **Disease duration (year)**  Mean ± SD | 12 ± 5·5 |
| **Previous treatments**  Adalimumab  Steroids  Methotrexate  Mycophenolate mofetil  Cyclosporine  Hydroxychloroquine  Azathioprine | 73% (n=8/11)  91% (n=10/11)  55% (n=6/11)  45% (n=5/11)  36% (n=4/11)  9% (n=1/11)  9% (n=1/11) |
| **Duration of Remicade^®^ treatment (year)**  Mean ± SD | 3·3 ± 3·3 |

**Supplementary table 4. Behcet’s disease activity measured with BDCAF* on Remsima^®^ therapy**

| **Patient** | **Gender** | **Behcet disease activity scores**  **Baseline 3^rd^ month 6^th^ month** | | |
| --- | --- | --- | --- | --- |
| 1· | M | 2 | 1 | 1 |
|  | V | 4 | 3 | 3 |
|  | V | 6 | 5 | 4 |
|  | V | 2 | 2 | 1 |
|  | M | 3 | 3 | 3 |
|  | M | 0 | 0 | 0 |
|  | M | 0 | 1 | 0 |
|  | M | 3 | 3 | 3 |
|  | M | 3 | 2 | 6 |
|  | V | 2 | 1 | 1 |
|  | M | 1 | 0 | 0 |
|  | V | 2 | 2 | 2 |

* **Behcet’s disease current activity form**

**Supplementary table 5. Uveitis disease activity measured with UVEDAI* on Remsima^®^ therapy**

| **Patient** | **Gender** | **Uveitis disease activity index**  **Baseline 3^rd^ month 6^th^ month** | | |
| --- | --- | --- | --- | --- |
| 1· | M | 2.12 | 1.70 | 1.49 |
|  | V | 0.42 | 0.84 | 1.22 |
|  | V | 2.37 | 1.91 | 1.91 |
|  | V | 1.89 | 1.26 | 1.64 |
|  | V | 1.53 | 2.58 | 2.16 |
|  | V | 1.74 | 1.32 | 1.32 |
|  | M | 0.69 | 0.69 | 0.69 |
|  | M | 6.23 | 5.81 | 4.55 |
|  | V | 0.69 | 0.77 | 0.9 |
|  | V | 1.11 | 1.53 | 1.11 |
|  | V | 0.71 | 0.98 | 1.28 |

* **Uveitis disease activity index**
